# Cisplatin-resistant A549 non-small cell lung cancer cells can be identified by increased mitochondrial mass and are sensitive to pemetrexed treatment

**DOI:** 10.1186/s12935-019-1037-1

**Published:** 2019-11-29

**Authors:** Yanyun Gao, Patrick Dorn, Shengchen Liu, Haibin Deng, Sean R. R. Hall, Ren-Wang Peng, Ralph A. Schmid, Thomas M. Marti

**Affiliations:** 1 Department of General Thoracic Surgery, Inselspital, Bern University Hospital, Murtenstrasse 50, 3008 Bern, Switzerland; 2 Department of BioMedical Research, University of Bern, Bern, Switzerland; 3 Department of Intensive Care Medicine, Inselspital, Bern University Hospital, Bern, Switzerland

**Keywords:** Lung cancer, NSCLC, Chemotherapy, Cisplatin, Pemetrexed, Resistance, Mitochondrial activity, Mitochondrial mass

## Abstract

**Background:**

Cisplatin plus pemetrexed combination therapy is considered the standard treatment for patients with advanced, non-squamous, non-small-cell lung cancer (NSCLC). However, advanced NSCLC has a 5-year survival rate of below 10%, which is mainly due to therapy resistance. We previously showed that the NSCLC cell line A549 harbors different subpopulations including a mesenchymal-like subpopulation characterized by increased chemo- and radiotherapy resistance. Recently, therapy resistance in hematological and solid tumors has been associated with increased mitochondrial activity. Thus, the aim of this study was to investigate the role of the mitochondrial activity in NSCLC chemotherapy resistance.

**Methods:**

Based on MitoTracker staining, subpopulations characterized by the highest 10% (Mito-High) or lowest 10% (Mito-Low) mitochondrial mass content were sorted by FACS (Fluorescence-Activated Cell Sorting) from paraclonal cultures of the NSCLC A549 cell line . Mitochondrial DNA copy numbers were quantified by real-time PCR whereas basal cellular respiration was measured by high-resolution respirometry. Cisplatin and pemetrexed response were quantified by proliferation and colony formation assay.

**Results:**

Pemetrexed treatment of parental A549 cells increased mitochondrial mass over time. FACS-sorted paraclonal Mito-High cells featured increased mitochondrial mass and mitochondrial DNA copy number compared to the Mito-Low cells. Paraclonal Mito-High cells featured an increased proliferation rate and were significantly more resistant to cisplatin treatment than Mito-Low cells. Interestingly, cisplatin-resistant, paraclonal Mito-High cells were significantly more sensitive to pemetrexed treatment than Mito-Low cells. We provide a working model explaining the molecular mechanism underlying the increased cisplatin- and decreased pemetrexed resistance of a distinct subpopulation characterized by high mitochondrial mass.

**Conclusions:**

This study revealed that cisplatin resistant A549 lung cancer cells can be identified by their increased levels of mitochondrial mass. However, Mito-High cells feature an increased sensitivity to pemetrexed treatment. Thus, pemetrexed and cisplatin target reciprocal lung cancer subpopulations, which could explain the increased efficacy of the combination therapy in the clinical setting.

## Background

Lung cancer is the most common cause of cancer-related mortality worldwide. This is mainly due to the difficulty of early detection and lack of effective treatment methods, thus more effective treatment options are desperately needed.

Standard therapy for NSCLC includes pemetrexed (MTA; commercial name ‘Alimta’), as single agent or in combination with either chemo-or radiotherapy [1]. MTA is a folic acid antagonist and works by inhibiting the synthesis of precursor purine and pyrimidine nucleotides and consequently DNA and RNA synthesis, thereby interfering with the proliferation and survival of replicating cancer cells. Human thymidylate synthase, the major target of MTA, is mainly localized in the mitochondria, which contain the complete pathway to conduct de novo thymidylate biosynthesis [2]. In this context, the expression of genes involved in pyrimidine metabolism, which are targeted by antifolates such as MTA, is highly upregulated in NSCLC stem cells [3]. However, current strategies of MTA-based therapeutic regimens are challenged by relapse of the disease [1].

Mitochondrial metabolism is essential for tumorigenesis and cancer cell proliferation [4]. It was shown before that a subpopulation of breast cancer stem-like cells can be identified by increased mitochondrial mass, which is associated with chemotherapy resistance [5]. In NSCLC, high mitochondrial activity correlated with increased sphere formation capacity and tumor growth, which is in agreement with the findings in pancreatic and breast cancer [5–8]. Treatment with various DNA damage-inducing agents affects mitochondrial mass and activity. In detail, treatment with the DNA damage-inducing agent doxorubicin increases mitochondrial mass over time in breast and colon cancer cells [9, 10]. It was shown that mitochondrial inhibitors in combination with chemotherapy can induce synthetic lethality in different cancer types [11–13].

Our recent study revealed that adaptation of the treatment schedule optimizes anticancer efficacy of MTA and cisplatin combination therapy and further revealed the existence of a chemotherapy resistant subpopulation in the NSCLC cell line A549 [14]. In a subsequent study [15], we showed that the parental, non-treated, A549 cell line contains phenotypically distinct subpopulations. In detail, holoclonal cells are characterized by an epithelial phenotype and feature a high tumor initiating capacity. In contrast, paraclonal cells feature a mesenchymal gene expression signature and give rise to fibroblast-like colonies characterized by the absence of intact colony borders. Interestingly, paraclonal cells were highly resistant to treatment with DNA damage-inducing agents but featured a low tumor initiating capacity. Indeed, it is well established that epithelial-mesenchymal transition (EMT) is also associated with chemotherapy resistance [16].

Despite the recognition that mitochondrial activity has a prominent role in nucleotide synthesis in cancer cells, its role in NSCLC chemotherapy resistance is relatively unknown. In this study, we take advantage of the paraclonal A549 subpopulation as a highly defined model to study how mitochondrial activity is associated with resistance to MTA and cisplatin, the components of the lung adenocarcinoma gold standard combination therapy. We found that cisplatin targets cells with a high mitochondrial activity. However, this subpopulation features an increased sensitivity to MTA treatment. Thus, our study revealed that MTA and cisplatin target different lung cancer subpopulations, which might explain the increased efficacy of the combination therapy in the clinical setting.

## Materials and methods

### Cell culture and media

The isolation of paraclonal subpopulations from the NSCLC cell line A549 (CCL-185) was described in detail in our previous publications [14, 15]. Purified A549 paraclonal cultures (subclone 3.4) were cultured in Dulbecco’s modified Eagle’s medium nutrient mixture F-12 Ham (DMEM/F12), supplemented with 9% fetal bovine serum, 1% penicillin/streptomycin solution and 1% l-Glutamine at 37 °C in a humidified 5% CO_2_ incubator. Cell lines were DNA fingerprinted as described previously [14, 15].

### Immunofluorescence microscopy

Four thousand A549 cells were seeded into 4-well chamber slides, each well containing 1 mL culture medium. After 2–3 days of culture, medium was removed and attached cells were washed with PBS for 5 min at room temperature. PBS was replaced by 1 mL of DMEM/F12 medium containing 200 nM MitoTracker Deep Red. Cells were incubated at 37 °C for 30 min and subsequently washed with 1 mL PBS. Cells were fixed with 0.5 mL FIX/PERM solution for 15 min at room temperature. After fixation, cells were washed with PBS and subsequently mounted with mounting media containing DAPI. Fluorescence microscopy images were acquired on a Leica DMI4000 microscope.

### Flow cytometry

A549 cells were seeded into 15-cm dishes (1 × 10^6^ cells per dish) on day 0. On day 1, MTA was added at a final concentration of 1 µM and plates were incubated at 37 °C as indicated for either 24, 48 or 72 h, respectively. Subsequently, 0.5 × 10^6^ cells were stained in culture medium with 200 nM MitoTracker Deep Red for 30 min at 37 °C. Cells were washed once with PBS and fixed by FIX/PERM solution. After fixation, cells were suspended in FC buffer (PBS with 2% FBS, 0.05% NaN_3_, 2 mM EDTA) containing 0.5 µg/mL DAPI and analysed using a FACS LSR2 flow cytometer.

### Western blot analysis

A549 cells were seeded into 6-well plates (0.15 × 10^6^ cells per well) and treated with MTA as described above. Subsequently, cells were lysed in RIPA buffer containing 1 × protease and phosphatase inhibitor cocktails. Protein concentration was measured by Pierce™ BCA Protein Assay Kit. Equal amounts of protein lysates (21 µg/lane) were resolved by SDS-PAGE, and transferred onto nitrocellulose membranes, which was then blocked in Intercept^®^ (TBS) Blocking Buffer for 1 h at room temperature and blotted with specific primary antibodies at 4 °C overnight on an orbital shaker. IRDye 680LT-conjugated goat anti-mouse IgG and IRDye 800CW-conjugated goat anti-rabbit IgG from Li-COR Biosciences were used at 1:5000 dilutions. Finally, signals of membrane-bound secondary antibodies were imaged and quantified using the Image Studio Lite System.

### Titration and toxicity analysis of MitoTracker dyes

A549 Rho0 cells were generated by continuous exposure for 3 month to ethidium bromide (50 ng/mL) as described previously [17]. Duplex PCR was used to validate the phenotype of A549 Rho0 cells [18]. Parental A549 and A549 Rho 0 cells were stained with MitoTracker Red or MitoTracker Deep Red at increasing concentrations. Stained cells were analysed by flow cytometry as described above. To evaluate toxicity, A549 paraclonal cells were stained with MitoTracker Deep Red at increasing concentrations and subsequently seeded into 6-well plates (5000 cells/well). After 1-week, cells were stained with crystal violet (1% in 50% ethanol).

### Fluorescence activated cell sorting (FACS)

1 × 10^6^ paraclonal A549 cells were seeded into 15 cm dishes. Once 70–80% confluence was reached, i.e. after 2 or 3 days, exponentially dividing paraclonal A549 cells were detached by TrypLE and re-suspended into cultured medium. Trypan blue staining was used to exclude dead cells and to determine cell titers. Approximately 80 × 10^6^ paraclonal cells were stained with 25 nM MitoTracker Deep Red for 30 min in cell culture medium at 37 °C. After staining, cells were washed once with PBS for 5 min. Subsequently, cells were resuspended in 5 µM Vybrant^®^ DyeCycle™ Violet staining solution (in PBS with 2%FBS). ARIA III (FACS Diva 8.0.1, BD). Finally, two regions containing each 10% of the total cell single cell population, either with the highest or lowest MitoTracker signal intensity, i.e. “Mito-High” and “Mito-Low” subpopulations, respectively, were FACS sorted using an ARIA III cell sorter (FACS Diva 8.0.1, BD). The detailed FACS gating strategy is depicted in Fig. 3.

### Real-time PCR (qPCR)

Sorted cells (Mito-High 10% and Mito-Low 10%) were washed with PBS. DNA genome was isolated and purified with GenElute™ Mammalian Genomic DNA Miniprep Kit. qPCR analyses were performed in triplicate on a 7500 Fast Real-Time PCR System (Applied Biosystems) with a new fluorescent DNA-binding dye-based (similar with SyberGreen I) GoTaq^®^ qPCR Master Mix. qPCR cycling parameters are used as follows: 10 µL-reaction system, Initial denaturation 95 °C, 2 min, 1 cycle; Denaturation 95 °C, 3  s, Annealing extension 68 °C, 30  s, 40 cycles. Relative quantification (mtDNA:nDNA ratio) was calculated using the ΔΔCt method upon targeting of nuclear-encoded genes(human β-globin_Fwd: 5′-GGCTGTCTCCTAGCAACGAC-3′, human β-globin_Rev: 5′-TGCATACCAGCTCTCACCTG-3′ and mitochondrial genome (221 bp human mitochondrial genome fragment_Fwd: 5′-CCC CAC AAA CCC CAT TAC TAA ACC CA-3′, human mitochondrial genome fragment_Rev: 5′-TTT CAT CAT GCG GAG ATG TTG GAT GG-3′) [19].

### High-resolution respirometry

High-resolution respirometry was performed on sorted Mito-High and Mito-Low subpopulations. In detail, approximately 1 × 10^6^ cells were resuspended in 2 mL of mitochondrial respiration medium (110 mM sucrose, 0.5 mM EGTA, 3.0 mM MgCl_2_, 80 mM KCl, 60 mM K-lactobionate, 10 mM KH_2_PO_4_, 20 mM taurine, 20 mM hepes, 1.0 g/l BSA, pH 7.1). Sorted subpopulations were analyzed at 37 °C using an Oroboros^®^ oxygraphy tool (Oxygraph-2 k, Oroboros Instruments, Innsbruck, Austria), with chamber volumes set at 2 ml as published previously by our group [20]. Basal coupled endogenous respiration of intact cells (oxygen consumption without the addition of exogenous substrate) was measured and recorded using the linear rate of oxygen consumption. DatLab software was used for data acquisition and analysis, which included calculation of the time derivative for oxygen concentration and correction for instrumental background oxygen flux.

### Cisplatin and MTA response assays

To quantify cell numbers after chemotherapy treatment, paraclonal A549 cells were stained for FACS sorting as described above. Subsequently, 10,000 Mito-High (10%) or Mito-Low (10%) cells per well were FACS-sorted into 6-well plates containing 2 mL culture medium with different concentrations of cisplatin (0, 0.25, 0.5, 1, 2, 4 µM) and MTA (0, 3.125, 6.25, 12.5, 25, 50 µM). Cells were cultured at 37 °C in a humidified 5% CO_2_ incubator. After 1 week, cells from three wells per treatment were harvested using ThypLE. Cell titers were determined using a hemocytometer and trypan blue (final concentration 0.1%) for dead cell exclusion. Experiments were repeated independently three times.

To quantify colony numbers after chemotherapy treatment, 700 cells per well were FACS-sorted into 6-well plates and treated with chemotherapy as described above. After 2 weeks, colonies were stained by crystal violet solution (1% final concentration in 50% ethanol). Colony formation was quantified by an automated colony counting system (GelCount, Oxford Optronix).

### Statistics

Statistical analyses were performed using GraphPad Prism 6.03 (GraphPad Software Inc., http://www.graphpad.com) unless otherwise indicated. In all studies, data represent biological replicates (n) and are depicted as mean values ± s.d. or mean values ± SEM as indicated in the figure legends. Comparison of mean values was conducted with two-tailed Student’s t test and two-way ANOVA with Tukey’s multiple comparisons test as indicated in the figure legends. In all analyses, p values less than 0.05 were considered statistically significant.

Additional information and resources are included in the Additional file 1: Table S1.

## Results

### MitoTracker Deep Red dye localizes to the mitochondria and confers toxicity at higher doses

We established an experimental approach to investigate whether mitochondrial activity is associated with chemotherapy resistance. In detail, we previously showed that distinct subpopulations co-exist in the parental NSCLC cell line A549, which are characterized by significant differences in EMT status [15]. To exclude the effect of EMT-associated parameters, we focused our analysis to one subpopulation only, A549 paraclonal cells, which are highly resistant to DNA-damaging therapy (Additional file 2: Figure S1a).

To obtain a negative control to assay mitochondrial activity, we depleted mitochondrial DNA from parental A549 cells (A549 Rho 0 cells) by continuous ethidium bromide treatment as published previously [17]. Indeed, whereas mitochondrial DNA was readily detectable in parental A549 cells, no mitochondrial DNA was detectable in A549 Rho 0 cells (Additional file 2: Figure S1b). In agreement, high-resolution respirometry revealed that only background activity of the mitochondrial respiration complex I, II, and IV (encoded by mitochondrial DNA) was detectable in A549 Rho 0 cells (Additional file 2: Figure S1c). Analysis by light microscopy revealed that the mitochondrial DNA ablation protocol induced obvious morphological changes (Additional file 2: Figure S1d), i.e. cells became larger and more mesenchymal like. Our analysis by flow cytometry revealed that the basal autofluorescence levels in the PE-Texas Red Channel of A549 Rho 0 cells were tenfold higher compared to parental A549 cells (Additional file 3: Figure S2, compare unstained signals, i.e. red peaks). This increase in autofluorescence of Rho 0 cells might be explained due to the supplementation of the growth medium with ethidium bromide, whose absorbance and emission spectra are a near-perfect match with those of PE-Texas Red (Additional file 3: Figure S2b, https://www.thermofisher.com/order/spectra-viewer).

MitoTracker dyes are frequently used to quantify mitochondrial activity in cancer cells (reviewed in [21]). According to the manufacturer, MitoTracker Red CMXRos is a fixable red-fluorescent dye that stains mitochondria in live cells and its accumulation is dependent on the mitochondrial membrane potential. However, when we stained A549 Rho 0 with the Rosamine-based dye MitoTracker RED CM-H2Ros at a concentration of >1 nM, average signal intensity increased more than tenfold compared to non-stained cells (Additional file 3: Figure S2). Therefore, we concluded that MitoTracker RED CM-H2Ros is not a suitable dye to monitor mitochondrial activity in A549 cells under our experimental conditions.

**Fig. 1:**
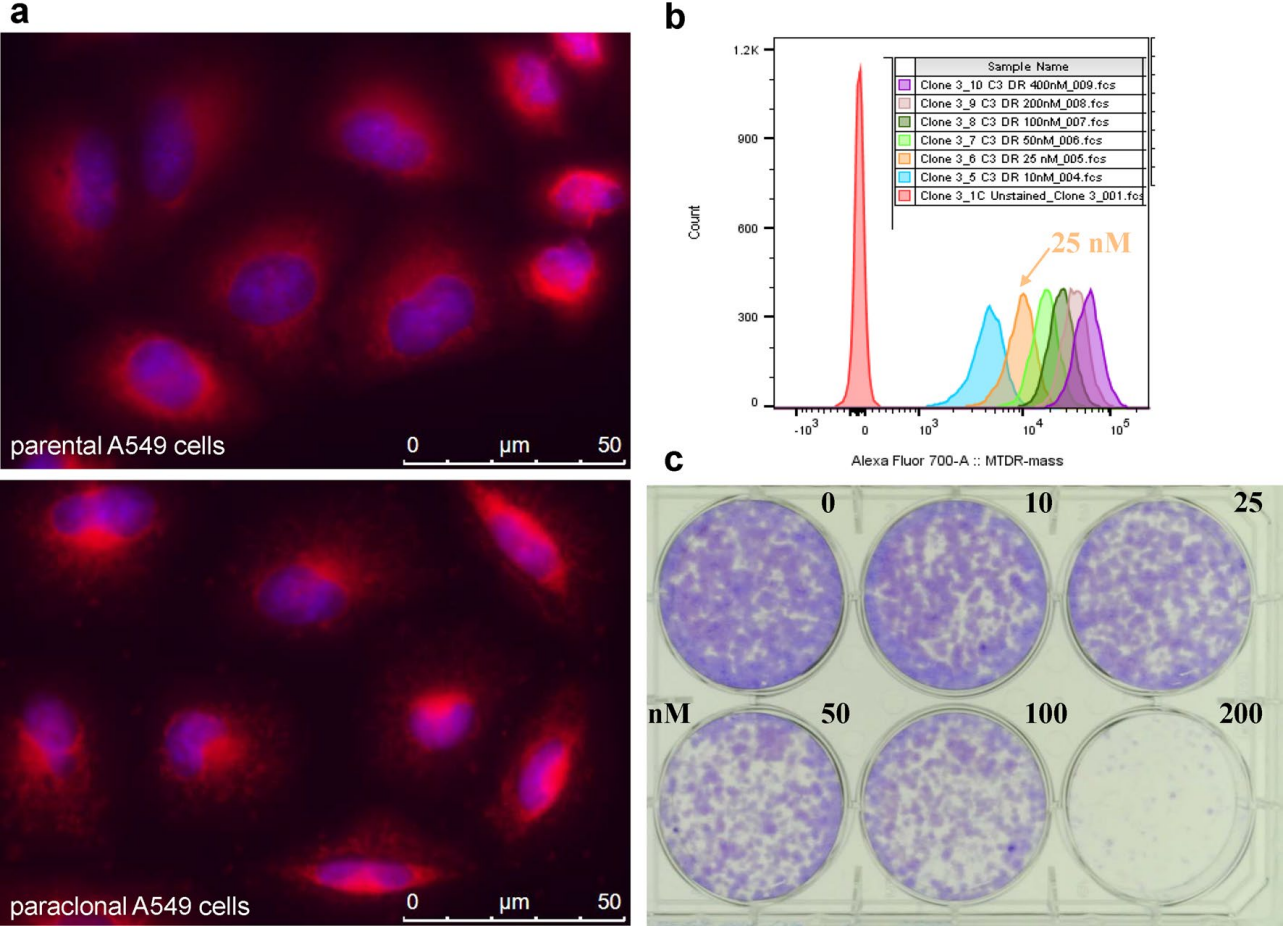
Localization, titration and toxicity of MitoTracker Deep Red staining in A549 cells. **a** Localization of MitoTracker Deep Red staining (red) in parental (top) and paraclonal A549 cells (bottom) analyzed by immunofluorescence microscopy. Cells were counterstained with DAPI. **b** Titration of MitoTracker Deep Red staining. Flow cytometry-based analysis of signal intensity from mesenchymal paraclonal A549 cells after staining with the indicated concentrations of MitoTracker Deep Red dye. **c** Assessing toxicity of MitoTracker Deep Red staining by colony formation assay. Colonies were stained by crystal violet 2 weeks after treatment with MitoTracker Deep Red dye at the indicated concentrations, e.g. 0, 10, 25, 50, 100 and 200 nM

In breast cancer, increased mitochondrial mass is associated with increased, sphere/tumor formation capacity and chemotherapy resistance [5]. Staining with the mitochondrial mass specific dye MitoTracker Deep Red resulted in a perinuclear staining pattern indicative of a mitochondrial localization in both, parental and paraclonal A549 cells (Fig. 1a). Analysis by flow cytometry revealed that MitoTracker Deep Red signal intensity increased in a dose dependent manner (Fig. 1b). However, higher concentrations of MitoTracker Deep Red decreased colony formation capacity of paraclonal A549 cells (Fig. 1c). Therefore, MitoTracker Deep Red was used at a concentration of 25 nM for staining cells in all subsequent experiments.

### MTA treatment increases mitochondrial mass over time

**Fig. 2:**
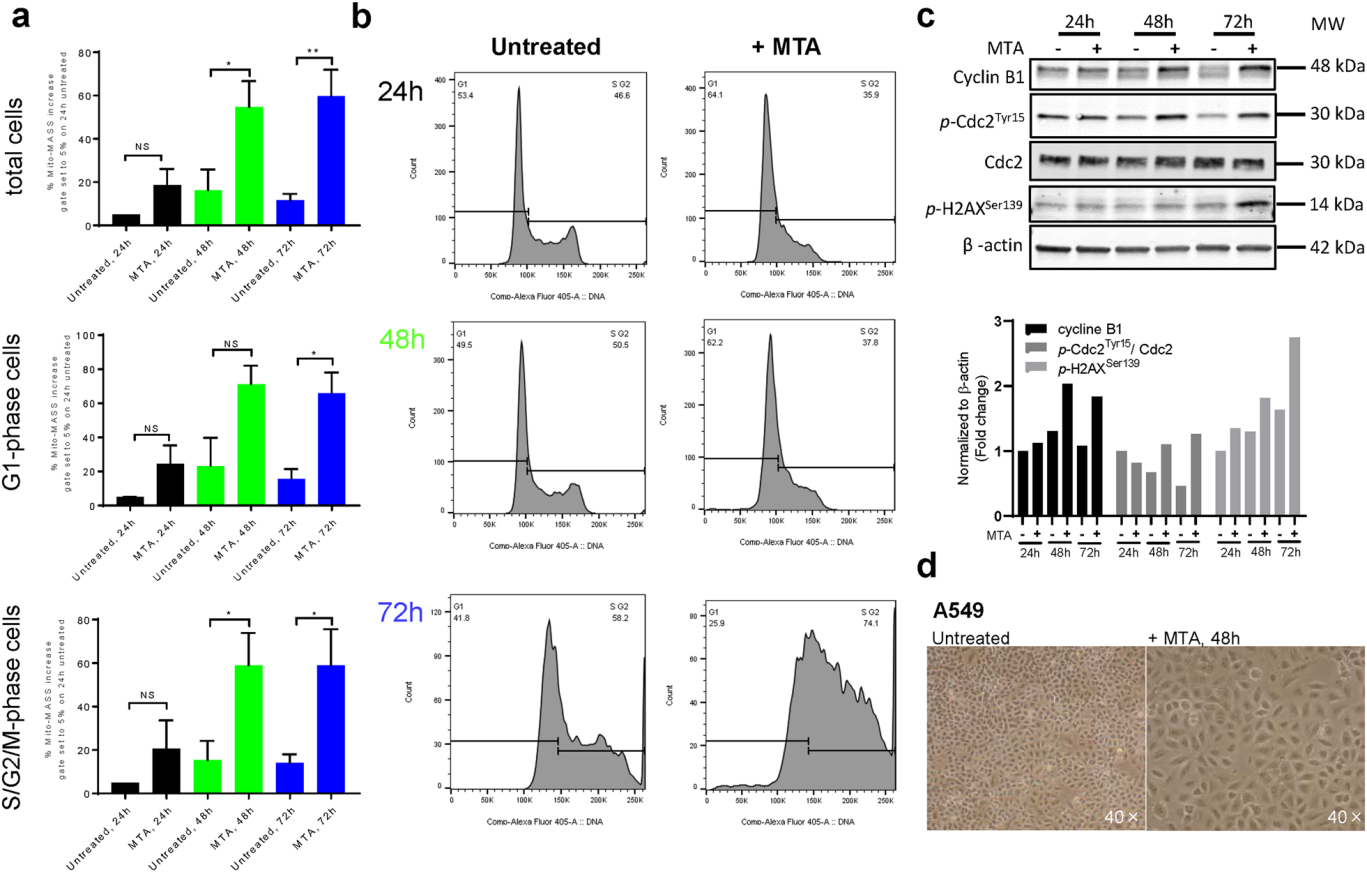
MTA treatment increases mitochondrial mass of lung cancer A549 cells. **a** Changes in mitochondrial mass after treatment with 1 µM MTA were quantified by FACS analysis. In detail, to quantify changes in mitochondrial mass, a gate was set so 5% of untreated A549 cells (24 h) were scored as positive for mitochondrial mass (see Additional file 4: Figure. S3 for details). The same gate was applied to all samples. The total population of cells was further divided into two subpopulations according to their respective DNA content, e.g. G1-phase cells and S/G2/M-phase cells. Shown are the mean values and the standard error of mean, N = 4. *p* value was determined by unpaired and two-tailed Student’s t test, *p < 0.05, **p = 0.0076. **b** Cell cycle distribution after MTA treatment. G1- and S/G2/M-phase gates were adjusted for each sample to compensate for slight shifts in linear DAPI fluorescence intensity due to treatment-induced changes in FSC/SSC signal intensity. **c** Analysis of parental A549 cell protein expression after MTA treatment by western blot. G_2_M cell cycle checkpoint proteins: Cyclin B1, Cdc2 and *p*-Cdc2^Tyr15^. DNA damage response protein: *p*-H2AX^Ser139^. Signal intensities were normalized to the internal control (β-actin) on the same blot. The control condition, i.e. untreated A549 cells (24 h), was set to unity. **d** MTA treatment for 48 h induced morphological changes in parental A549 cells

We investigated whether MTA treatment increases mitochondrial mass in NSCLC A549 cells. Indeed, treatment of parental A549 cells for 24 h with 1 µM MTA increased mitochondrial mass, which was further augmented after 48 and 72 h, respectively (Fig. 2a, see Additional file 4: Figure S3a for the gating strategy). Additionally, these results further corroborated our findings that staining with MitoTracker Deep Red is suitable to monitor relative changes in mitochondrial mass.

Mitochondrial content and cell size both increase during progression through the cell cycle. Consequently, mitochondrial content has to be normalized to the cell cycle phase, as described in the literature before [22]. Therefore, we established a flow cytometry protocol, which included simultaneous staining for mitochondrial mass and DNA content (Additional file 4: Figure S3b). MTA treatment for 24 h induces a G1/S-phase arrest in parental A549 cells (Fig. 2b). The G1-phase arrest was further augmented by MTA treatment for 48 h, as we published before [14, 23]. This indicates that the observed increase in mitochondrial mass after treatment with 1 µM MTA is not sufficient to overcome the induction of a cell cycle arrest. In this study, we additionally observed a transition into the later cell cycle phases, e.g. S/G2/M-phase, 72 h after starting the treatment with MTA (Fig. 2b). The finding that 72 h of MTA treatment induces a robust G_2_/M-phase cell cycle arrest by was further corroborated by the accumulation G_2_/M cell cycle checkpoint proteins, i.e. Cyclin B1 and phosphorylated Cdc2, (reviewed in [24]). We previously showed that 48 h of MTA treatment induces DNA damage as indicated by increased levels of phosphorylated H2AX [14, 23]. Indeed, levels of H2AX phosphorylation after MTA treatment were further increased after 72 h compared to 48 h Fig. 2c). Interestingly, we observed that the increase in cellular mitochondrial mass at 48 and 72 h after the start of the MTA treatment was detectable in both G1-phase and S/G2/M-phase cells, respectively. Hence, the increase in mitochondrial mass after MTA treatment is independent of the cell cycle phase (Fig. 2a, middle and bottom panel).

### A549 subpopulations isolated based on mitochondrial mass differ in mitochondrial DNA content and basal respiratory activity

**Fig. 3:**
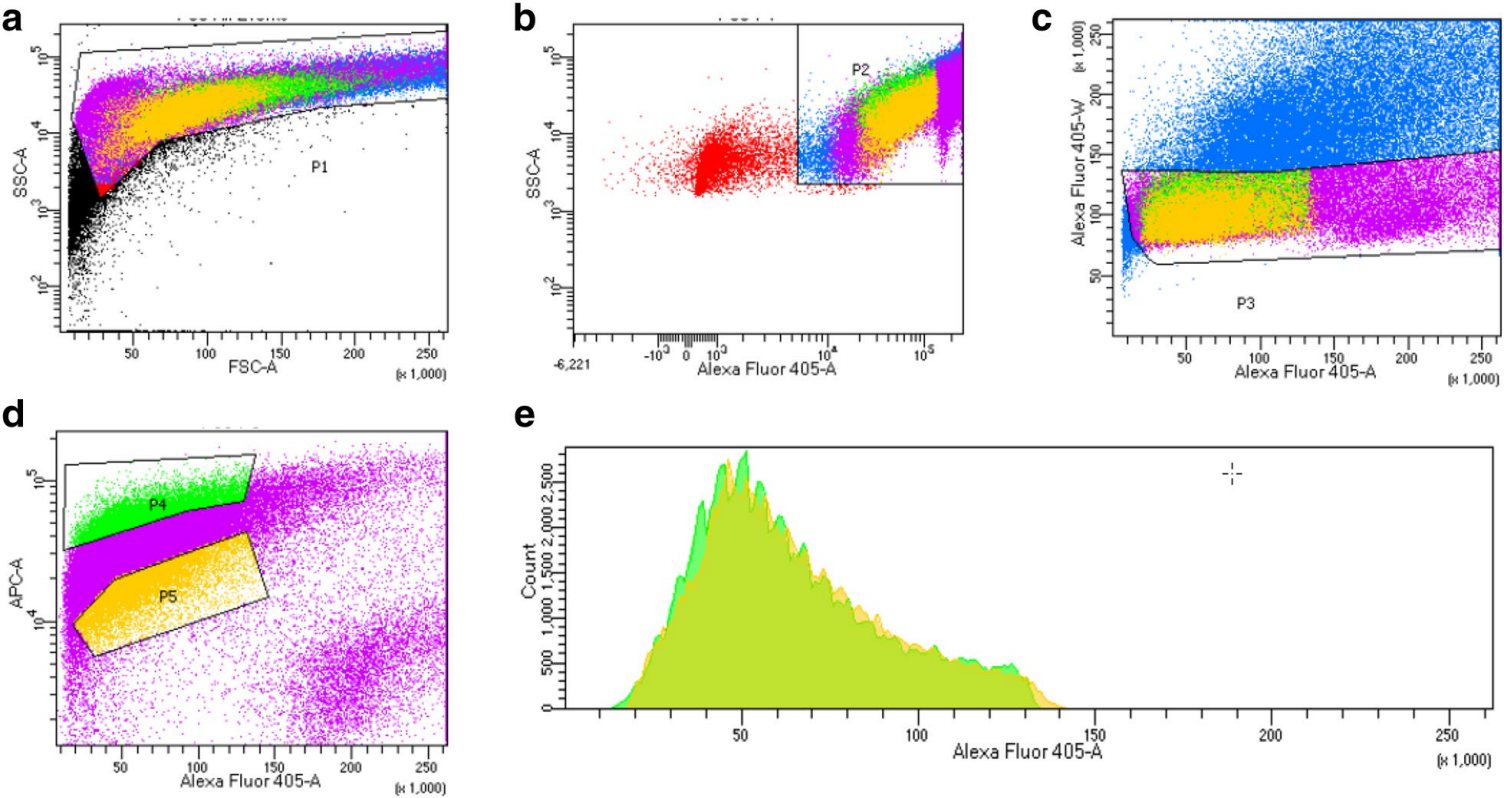
FACS-based isolation of Mito-High and Mito-Low subpopulations from paraclonal A549 cells. For cell sorting, the following gates were sequentially applied: **a** Debris exclusion based on the cell size and granularity (FSC-A vs SSC-A, respectively), e.g. gate P1 includes 96.4% of total population. **b** Second round of debris exclusion based on DNA content (Alexa Fluor 405-A, e.g. gate P2 includes 97.1% of gate P1. **c** Single cell gate based on DNA content measurement (Alexa Fluor 405-A vs Alexa Fluor 405-W, e.g. gate P3 includes 73.6% of gate P2. **d** Based on MitoTracker Deep Red staining (APC-a), sorting gates P4 and P5 were defined so each contains 10% of the total single cell population, either with the highest or lowest MitoTracker signal intensity, i.e. “Mito-High” and “Mito-Low”, respectively

To exclude the effect of EMT-associated parameters, we limited our analysis to A549 paraclonal cells, which are highly resistant to DNA-damaging therapy (Additional file 2: Figure S1a). We developed a FACS sorting strategy to isolate subpopulations based on their differential MitoTracker Deep Red staining intensities (Fig. 3). In detail, staining for DNA content allowed us to exclude debris (Fig. 3a, b) and doublets (Fig. 3c). Finally, we gated for two regions containing each 10% of the total cell single cell population, either with the highest or lowest MitoTracker signal intensity, i.e. “Mito-High” and “Mito-Low” subpopulations, respectively (Fig. 3d).

We adjusted the layout of our sorting gates so the cell cycle distribution of the sorted populations was not only similar with the non-sorted A549 paraclonal population but also matched each other (Fig. 3e). Staining of live cells with Hoechst results in toxicity at higher concentrations; therefore, we used a low Hoechst concentration for our FACS protocol. The cell cycle distribution histograms obtained by Hoechst staining during our live-cell sorting protocol did not feature the characteristic G1, S and G2/M phases (Fig. 3e), which we observed after DAPI staining of fixed and permeabilized cells (Fig. 2b). However, the live-cell Hoechst staining nevertheless allowed us to match the cell cycle distribution of the sorted subpopulations by adjusting the sorting gates accordingly (Fig. 3e).

**Fig. 4:**
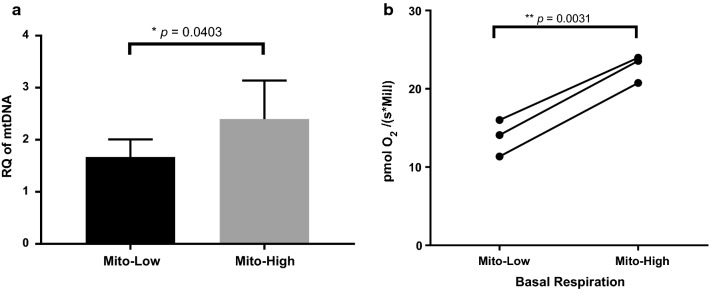
Characterization of the sorted Mito-High and Mito-Low subpopulations from paraclonal A549 cells. **a** Relative quantification of mtDNA content by qPCR, *p* value was determined by two-tailed Student’s t test, *p = 0.0403 **b** Basal respiration (O_2_ consumption rate) measured by high-resolution respirometry (OROBOROS). Y axis showed the O_2_ consumption rate, *p* value was determined by paired Student’s t test, *p *= 0.0031

Our subsequent analysis of the sorted subpopulations revealed that the mitochondrial DNA content and basal respiration (indicator of mitochondrial function) was significantly higher in the Mito-High compared to the Mito-Low subpopulation, respectively (Fig. 4). In summary, we developed a sorting strategy based on mitochondrial mass, which allowed us to isolate from paraclonal A549 cultures distinct subpopulations characterized by significant differences in mitochondrial DNA copy numbers and activity.

### High mitochondrial mass is associated with increased cellular proliferation and cisplatin resistance

**Fig. 5:**
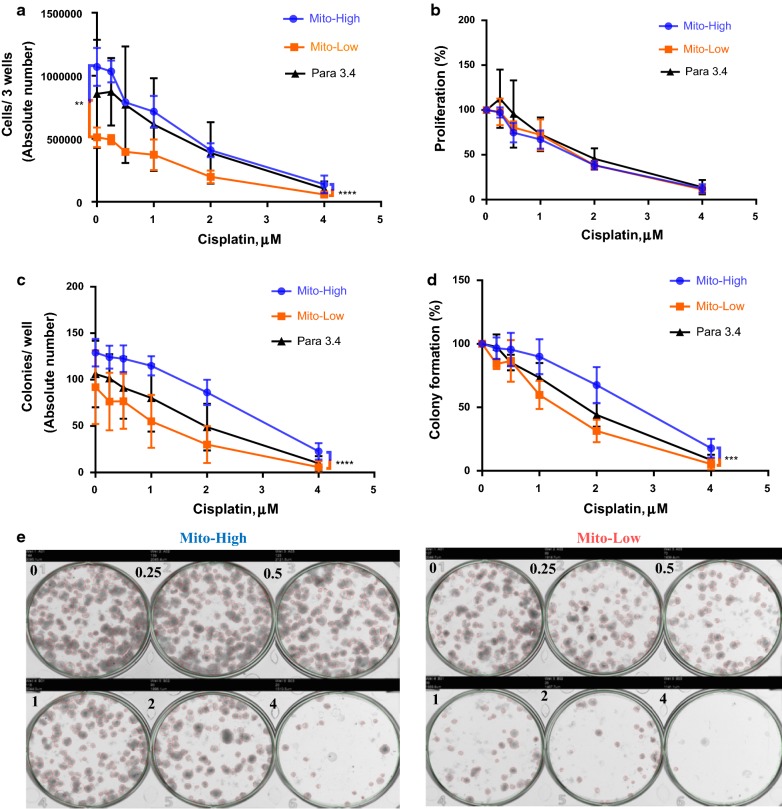
High mitochondrial mass is associated with increased cellular proliferation and cisplatin resistance. Mito-High: highest 10% of Mito Tracker stained cells; Mito-Low: lowest 10% of Mito Tracker stained cells. Para 3.4: unsorted Para clone cells (the subtype is 3.4). **a** Absolute cell numbers per well 1 week after cisplatin treatment at the indicated concentrations. **b** Normalized cell growth based on the cell numbers without cisplatin treatment, i.e. untreated cell numbers (**a**) were set as 100%. **c** Absolute colony numbers per well 2 weeks after cisplatin treatment at the indicated concentrations. **d** Normalized colony formation capacity based on colony numbers without cisplatin treatment, i.e. untreated colony numbers (**c**) were set as 100%. **e** Images of colony formation for two populations Mito-High (left) and Mito-Low (right)

Cellular proliferation, i.e. cell numbers 1 week after sorting in the absence of treatment, was significantly increased in the Mito-High compared to the Mito-Low paraclonal A549 subpopulation (Fig. 5a). Additionally, absolute cell numbers were also higher after cisplatin treatment at different doses (Fig. 5a). However, after adjusting for the basal difference in proliferation, relative inhibition of proliferation by cisplatin treatment was identical for Mito-Low and Mito-High paraclonal A549 subpopulations (Fig. 5b).

In tumors, eradication of the capacity for unlimited proliferation of all stem cells is required for the prevention of recurrences [25]. In vitro, the clonogenic assay is the method of choice to test for the capacity of a cell to undergo “unlimited” division and thus has been used as a surrogate assay to evaluate tumor initiation capacity and stem cell status [26]. Indeed, in the absence of treatment, absolute colony numbers from Mito-High subpopulations were significantly higher compared to the colonies formed after seeding Mito-Low paraclonal A549 cells (Fig. 5c, e). Additionally, absolute colony numbers were also higher after cisplatin treatment at different doses. In contrast to the results obtained by analyzing cell numbers, after adjusting for the basal difference in colony formation, the colony formation capacity of Mito-High cells was significantly more resistant to cisplatin treatment compared to Mito-Low cells (Fig. 5d).

### High mitochondrial mass is associated with increased MTA sensitivity

**Fig. 6:**
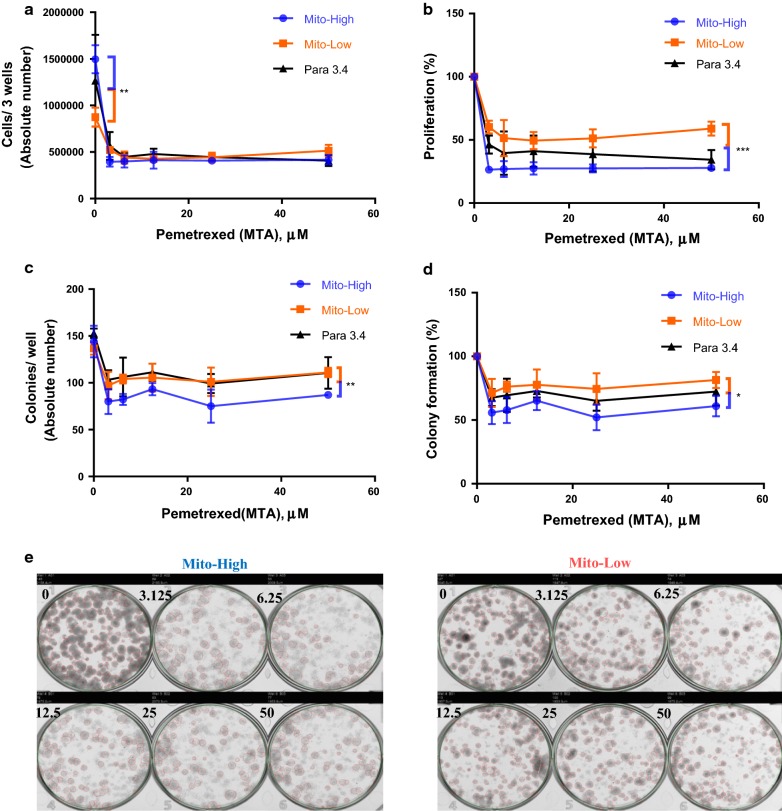
High mitochondrial mass is associated with increased MTA sensitivity. The cell population with high mitochondrial mass is sensitive to pemetrexed (MTA). Mito-High: highest 10% of Mito Tracker stained cells; Mito-Low: lowest 10% of Mito Tracker stained cells. Para 3.4: unsorted Para clone cells (the subtype is 3.4) **a** Absolute cell numbers per well 1 week after MTA treatment at the indicated concentrations. **b** Normalized cell growth based on the cell numbers without MTA treatment, i.e. untreated cell numbers (**a**) were set as 100%. **c** Absolute colony numbers per well 2 weeks after MTA treatment at the indicated concentrations. **d** Normalized colony formation capacity based on colony numbers without MTA treatment, i.e. untreated colony numbers (**c**) were set as 100%. **e** Images of colony formation assay for the two populations Mito-High (left) and Mito-Low (right)

Cisplatin plus MTA is the standard regimen for first-line treatment of advanced NSCLC [27]. Interestingly, both absolute and relative cellular proliferation of cisplatin-resistant Mito-High cells was significantly more affected by MTA treatment compared to Mito-Low cells (Fig. 6a, b). Similarly, both the absolute and the relative colony formation capacity of Mito-High cells were significantly more reduced by MTA treatment compared to Mito-Low cells (Fig. 6c–e, respectively).

In summary, our experiments indicate that MTA treatment increases mitochondrial mass in parental A549 NSCLC cells. Interestingly, relative to Mito-Low cells, Mito-High paraclonal A549 cells are resistant to cisplatin but sensitive to MTA treatment.

## Discussion

Chemotherapy resistance is a major obstacle in cancer therapy (reviewed in [28]). It was shown before that mitochondrial activity is associated with chemotherapy resistance in breast cancer [5]. In agreement, our study revealed that also in lung cancer, response to chemotherapy is dependent on the status of the mitochondrial metabolism.

Our study revealed that mitochondrial mass increased after MTA treatment (Fig. 3a). In agreement, in human colorectal adenocarcinoma cells, inhibition of nucleotide synthesis by treatment with the ribonucleotide reductase inhibitor Gemcitabine (2′,2′-difluorodeoxycytidine, a pyrimidine nucleoside analogue) results in a significant increase in mitochondrial mass quantified by MitoTracker Deep Red staining [29]. Recently, it was shown that the increase in mitochondrial mass in cancer cells after exposure to ionizing radiation is dependent on increased calcium accumulation in the mitochondria [30]. Previously, it was shown that the induction of mitochondrial biogenesis by etoposide is dependent on ATM and AMPK [31]. Thus, additional experiments will be required to clarify the underlying mechanism responsible for the increase in mitochondrial mass after MTA treatment in A549 lung cancer cells. Thus, it will be interesting to elucidate if the treatment-induced increase of mitochondrial mass in parental A549 cells (Fig. 2) is limited to Mito-Low cells or whether Mito-High cells can increase mitochondrial content to even higher levels after chemotherapy. Additionally, it was shown that cisplatin treatment can induce morphological changes, e.g. mitochondrial fusion [32]. Thus, it would be interesting to test whether chemotherapy not only induces changes in mitochondrial mass but also gives rise to functional and morphological changes.

The cancer stem cell hypothesis postulates that different cellular subpopulations do exist in tumors (reviewed in [33]). Cancer stem-like cells can self-renew and give rise to transient amplifying cells, which are responsible for bulk tumor cell proliferation. Quantifying tumor cell proliferation serves as an in vitro surrogate assay to quantify proliferation of bulk tumor cells whereas quantifying colony or sphere formation capacity serves as an in vitro surrogate assay to assess tumor-initiation capacity. Treatment with increasing doses of cisplatin reduces both, absolute and relative proliferation of all tested populations (Fig. 5a, b, respectively). Surprisingly, normalized proliferation of mito-Low and mito-High cells was equally inhibited by cisplatin treatment (Fig. 5b). This indicates that the effect of cisplatin on cellular proliferation is independent of the mitochondrial content. Alternatively, the increased absolute proliferation rate of mito-High cells might be more sensitive to cisplatin treatment thereby cancelling out the protective effect of an increased mitochondrial content.

Lung cancer tumor-initiating cells gave rise to chemotherapy-resistant colonies in vitro [34]. Also, breast cancer cells characterized by increased mitochondrial mass featured an increased tumor initiation capacity and gave rise to chemotherapy resistant colonies in vitro [5]. In agreement, we found that the absolute colony formation capacity of untreated Mito-High cells was increased compared to Mito-Low cells (Fig. 5c). Interestingly, the relative colony formation capacity of Mito-High cells was resistant to cisplatin treatment (Fig. 5d). This indicates that the effect of cisplatin on the relative colony formation capacity is dependent of mitochondrial content. In summary, untreated paraclonal A549 Mito-High cells feature an increased absolute proliferation rate and colony formation capacity. Interestingly, the relative effect of cisplatin on cellular proliferation might be independent of mitochondrial content whereas the effect of cisplatin on colony formation is dependent on the mitochondrial content. Further experiments will be required to elucidate the specific mitochondrial features rendering colony-forming cells resistant to cisplatin.

We found that the absolute proliferation rate of untreated Mito-High cells was increased compared to Mito-Low cells (Figs. 5a and 6a). In yeast, it was shown that dNTP pools are limiting for normal DNA replication [35]. In mammalian cells, the de novo pyrimidine biosynthetic enzyme dihydroorotate dehydrogenase (DHODH) is localized at mitochondria and is physically associated with the mitochondrial respiratory complex III [36]. Thus, we speculate that an increase in mitochondria-dependent nucleotide synthesis is responsible for the increased proliferation rate of Mito-High cells.

**Fig. 7:**
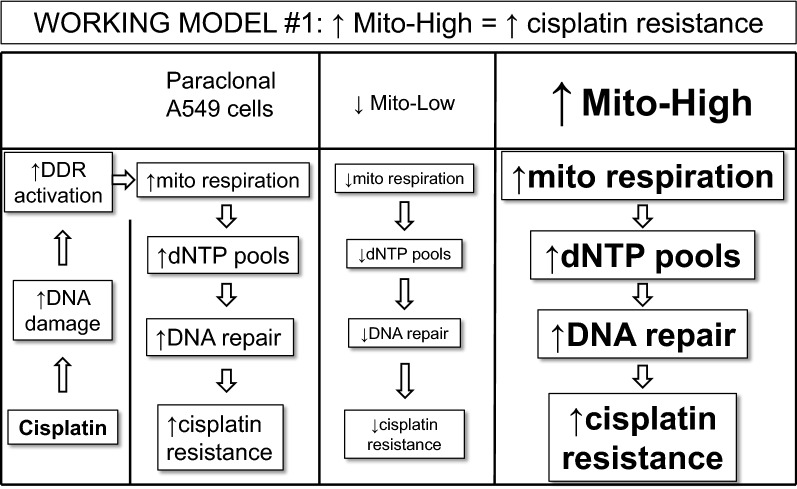
Working model explaining the increased cisplatin resistance of Mito-High cells. See text for details

Induction of DNA damage induces upregulation of dNTP levels in bacteria and yeast (reviewed in [37]). In detail, initiation of the DNA damage response activates respiration and thereby enlarging dNTP pools to promote cell survival in budding yeast [38]. In mammalian cells, the increase in nucleotide levels upon DNA damage induction is less pronounced than in single cell organisms (reviewed in [37]). However, in human breast cancer cells, treatment with the doxorubicin marginally increased purine nucleotide but significantly increased pyrimidine nucleotide levels [39]. Thus, we propose the following working model to explain the observed cisplatin resistance of Mito-High cells. DNA damage induction leads to the activation of the DNA damage response, which leads to a mitochondrial respiration-dependent increase of dNTP pools thereby increasing DNA repair capacity and and thus cisplatin resistance (Fig. 7). Mito-High cells contain more mitochondria per cell than Mito-Low cells, resulting in a relative increase in mitochondrial-dependent dNTP synthesis and subsequently increased DNA repair capacity, which could explain the increased cisplatin resistance of Mito-High cells (Fig. 7).

**Fig. 8:**
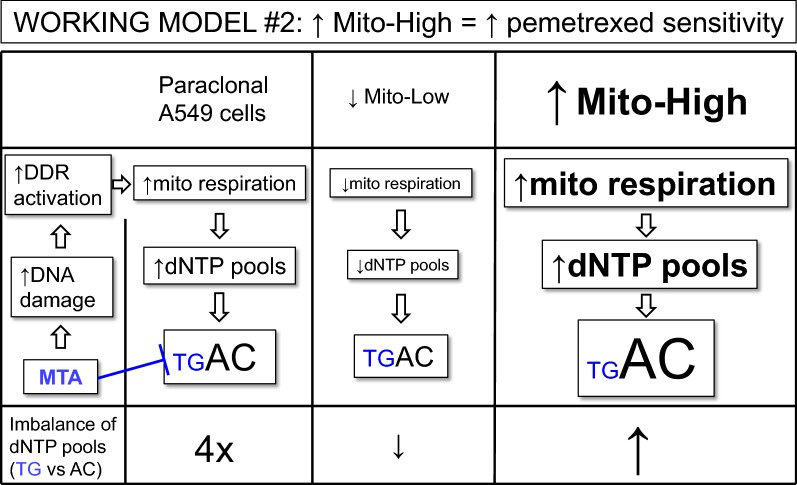
Working model explaining the increased MTA sensitivity of Mito-High cells. See text for details

Surprisingly, we found that cisplatin-resistant Mito-High cells featured an increased MTA sensitivity (Fig. 6). In contrast to cisplatin, MTA does not directly induce DNA damage. MTA blocks several enzymes involved in nucleotide synthesis, its main target being thymidylate synthetase, which is critical for the synthesis of dTMP [40]. MTA-induced nucleotide depletion will lead to DNA replication fork stalling and activation of the DNA damage response. Thus, according to our working model for cisplatin resistance (Fig. 7), we would conclude that Mito-High cells are more MTA resistant due to the relative increase in nucleotide levels. However, our experiments indicated the opposite, how can this be explained? As reviewed recently, not only are the overall dNTP levels important for genome stability, but also the balance between individual dNTPs since distortions in dNTP ratios can lead to DNA polymerase incorporation errors (reviewed in [37]. Thus, to explain the increased MTA sensitivity of Mito-High cells, we propose the following working model (Fig. 8), which is based on two assumptions: First, we assume that the dNTP pools are decreased in sorted Mito-Low and increased in Mito-High cells compared to paraclonal A549 cells. As discussed above, we speculate that the increased proliferation rate of Mito-High cells is due to an increased mitochondria-dependent nucleotide synthesis, i.e. increased dNTP pools. Second, we assume that MTA treatment reduces dGTP and dTTP levels in all cells four-fold compared to parental cells. Indeed, MTA treatment of HCT116 colorectal cancer cells reduced dGTP and dTTP levels by a factor of ~ fourfold compared to untreated controls wheras dATP and dCTP levels remained constant [41]. Thus, our working model predicts that the relative nucleotide pool imbalance will be most pronounced in Mito-High cells, which could explain the increased MTA sensitivity of Mito-High cells. Obviously, additional studies will be required to confirm the underlying molecular mechanisms of our working model. Additionally, it was shown before that ATP-binding cassette proteins are involved in cisplatin resistance [42]. Thus, further experiments will be required to determine if these proteins are associated with mitochondrial mass in the context cisplatin and MTA resistance.

The most significant limitation of our study is that the analysis is restricted to the A549 paraclonal subpopulation only. Our preliminary experiments revealed that sorting parental A549 cells according to mitochondrial mass staining for the top and bottom 5% subpopulations selects for paraclonal and holoclonal cells, respectively (data not shown). Thus, sorting cell lines for subpopulations according to mitochondrial mass does not address for differences in the EMT status. We previously showed that chemotherapy resistance is more than 100-fold higher in paraclonal compared to holoclonal cells [15]. Thus, isolated A549 subpopulations represent an ideal system to examine the association between mitochondrial mass and chemotherapy resistance without bias from the EMT status (Additional file 2: Figure S1a). Nevertheless, additional studies will be necessary to confirm that the observed association of mitochondrial activity with cisplatin resistance and MTA sensitivity is not limited to the highly resistant A549 paraclonal subpopulation only but applies to lung cancer cells in general. Additionally, quantifying mitochondrial dNTP pools would further corroborate our working models.

Accumulating evidence indicates that mitochondrial metabolism is required for tumorigenesis (reviewed in [4]. More recently, mitochondrial metabolism has been identified as a potentially fruitful target for cancer therapy. Indeed, several drugs targeting mitochondrial metabolism are currently under clinical investigation as novel anticancer therapies, either alone or as combination therapy (reviewed in [43]). Our study revealed that Mito-High cells are relatively resistant to cisplatin but sensitive to MTA treatment. Thus, targeting the mitochondrial metabolism might improve the anticancer activity of some drugs but might confer resistance for other agents. Therefore, it will be important to elucidate the exact molecular mechanism underlying any combination therapy targeting the mitochondrial metabolism. Hence, our study warrants further experiments to elucidate the critical role of mitochondrial metabolism in cancer growth and therapy resistance.

## Conclusion

Cisplatin resistant non-small cell lung cancer cells are characterized by high levels of mitochondrial mass and are susceptible to MTA treatment. Thus, MTA and cisplatin target reciprocal lung cancer subpopulations, which could explain the increased efficacy of the combination therapy in the clinical setting.

## Supplementary information

**Additional file 1: Table S1.** Key resources.

**Additional file 2: Figure S1. a** Working model to investigate the relationship between mitochondrial mass and chemotherapy resistance in subpopulations of the NSCLC cell line A549: We previously described three subpopulations in the parental cell line A549, e.g. holo-, mero- and paraclonal cells [16]. ChemoR (~ 100×) indicates that mesenchymal para clone cells are roughly 100 times more resistant to chemotherapy compared to holoclone cells. The question would be whether mitochondrial mass is correlated with chemoresistance (ChemoR (?x)). Within the mesenchymal paraclonal subpopulation, “A” and “B” indicate further subpopulations featuring either high or low mitochondrial mass, respectively. TICs: Tumor Initiating Cells. **b** Respiration measurement in A549 subpopulations and A549 Rho 0 cells: Duplex PCR products of A549 and A549 Rho 0 cells, mitochondrial DNA gene HVR (901 bp) nuclear DNA gene hNuc(467 bp). **c** Complex activity measurement of A549 and A549 Rho 0 cells by high-resolution respirometry OROBOROS dig, digitonin (for cell permeabilization), gm, glutamate and malate (providing nicotinamide adenine dinucleotide (NADH) to the respiratory chain complex I activation); adp, ADP (adenosine diphosphate); rot, rotenone (complex I inhibitor); succ, succinate (substrate of complex II); aa, antimycin A (Inhibitor of complex III); at, ascorbate and TMPD (N,N,N′,N′-tetramethyl-p-phenylendiamine) (substrate of Complex IV); az, sodium azide (Complex IV inhibitor). **d** Phase contrast images of A549 and A549 Rho 0 cells.

**Additional file 3: Figure S2.** Unspecific MitoTracker Red CMXRos staining. **a** Flow cytometry analysis of A549 and A549 Rho0 cells staining with MitoTracker Red CMXRos (Mitochondrial activity dye). Different concentrations of MitoTracker Red CMXRos dye were tested, e.g. 0.25, 0.5, 1, 5, 10, 25, 50 and 100 nM. **b** Absorbance and emission spectra of ethidium bromide and PE-Texas Red.

**Additional file 4: Figure S3.** Gating strategy to analyze the increase of mitochondrial mass after MTA treatment. **a** The gate mito-MASS^+^ was set as 5% in untreated A549 cells. **b** Mitochondrial mass distribution in different cell cycle phases of A549 cells. Mitochondrial mass of cells in G2 phase was 2 times higher comparing with G1 cells.

## Data Availability

Data sharing is not applicable to this article as no datasets were generated or analyzed during the current study.

## Abbreviations

NSCLC: non-small-cell lung cancer
Mito-High: FACS-sorted subpopulation characterized by the highest 10% mitochondrial mass content
Mito-Low: FACS-sorted subpopulation characterized by the lowest 10% mitochondrial mass content
MTA: multitargeted antifolate, pemetrexed
FACS: Fluorescence-Activated Cell Sorting
EMT: epithelial mesenchymal transition
ATM: ataxia telangiectasia mutated
AMPK: AMP (Adenosine monophosphate)-activated protein kinase
DDR: DNA damage response
