# Supplementary material for: Cisplatin-resistant A549 non-small cell lung cancer cells can be identified by increased mitochondrial mass and are sensitive to pemetrexed treatment
Source: Cancer Cell Int. 2019 Nov 29;19:317. doi: 10.1186/s12935-019-1037-1 (PMC6883680; doi:10.1186/s12935-019-1037-1)
Supplement: Supplementary file 1 — Additional file 1: Table S1. Key resources. [file 12935_2019_1037_MOESM1_ESM.docx]

Table S1 KEY RESOURCES

| REAGENT OR RESOURCE | SOURCE | IDENTIFIER | USAGE |
| --- | --- | --- | --- |
| **Antibodies** | | | |
| Anti- Cyclin B1 | Cell Signaling Technology | Cat. #4138S | 1:500 |
| Anti-*p*-Cdc2^Tyr15^ | Cell Signaling Technology | Cat. #9111S | 1:500 |
| Anti-Cdc2 | Cell Signaling Technology | Cat. #77055S | 1:500 |
| Anti-*p*-H2AX^Ser139^ | BioLegend | Cat. #613402 | 1:1000 |
| Anti-β-actin | Cell Signaling Technology | Cat. #3700S | 1:5000 |
| IRDye 800CW-conjugated goat anti-rabblit IgG | Li-COR Biosciences | Cat. #926-32211 | 1:5000 |
| IRDye 680LT-conjugated goat anti-mouse IgG | Li-COR Biosciences | Cat. #926-68020 | 1:5000 |
| **Drugs** | | | |
| Pemetrexed (MTA) | Elli Lilly | ALIMTA | - |
| Cisplatin | Sandoz | Cat. #44033792 | - |
| **Materials for Cell culture** | | | |
| Dulbecco’s modified Eagle’s medium nutrient mixture F- 12 Ham | Sigma-Aldrich | Cat. #D6421 | - |
| Dulbecco’s modified Eagle’s medium nutrient mixture F- 12 Ham High glucose | Sigma-Aldrich | Cat. #D6429 | - |
| fetal bovine serum | Life Technologies | Cat. #10270-106 | 9 % for cell culture |
| Penicillin/Streptomycin solution | Sigma- Aldrich | Cat. #P0781 | 1 % for cell culture |
| L-Glutamine | Sigma- Aldrich | Cat. #25030-024 | 1 % for cell culture |
| Thermo Scientific™ Nunc™ Lab-Tek™ II Chamber Slide™ System | Thermo Scientific Nunc | Cat. NNU♯154526 | - |
| UltraPure™ Ethidium Bromide | Thermo Fisher Scientific | Cat. #15585011 | 50 ng/mL |
| Sodium pyruvate | Sigma- Aldrich | Cat. #113246 | 1 mM |
| Uridine | Sigma- Aldrich | Cat. #58968 | 50 µg/mL |
| 10× TrypLE | Life Technologies, ThermoFisher | Cat. #A1217702 | 1× |
| 15 cm dishes | Bioswisstec | Cat. #20151 | - |
| 6-well plate | Falcon | Cat. #353046 | - |
| **Other Materials** | | | |
| MitoTracker® Deep Red FM | Invitrogen | Cat. #M22426 | 200 nM (IF), 25 nM (FACS) |
| MitoTracker® Red CM-H2XRos | Invitrogen | Cat. #M7513 | - |
| Vybrant® DyeCycle™ Violet Stain | Thermo Fisher Scientific | Cat. #V35003 | 5 µM |
| FIX/PERM solution | BD Bioscience | Cat. #554722 | 0.5 mL/ 1×10^6^ cells |
| Prolong Gold antifade reagent | Life technology | Cat. #P36931 | - |
| RIPA buffer | Cell Signaling Technology | Cat. #9806 | 50 µL/ well, 6-well plate |
| 100× protease and phosphatase inhibitor cocktail | Thermo Fisher Scientific | Cat. #78440 | 1× |
| Pierce™ BCA Protein Assay Kit | Thermo Fisher Scientific | Cat. #23227 | - |
| SDS-PAGE gel | Bio-Rad Laboratories | Cat. #4561095 | - |
| nitrocellulose membranes | Bio-Rad | Cat. #170-4158 | - |
| Intercept® (TBS) Blocking Buffer | Li-COR Biosciences | Cat. #927–60001 | - |
| GenElute™ Mammalian Genomic DNA Miniprep Kit | Sigma- Aldrich | Cat. #G1N350 | - |
| GoTaq® qPCR Master Mix | Promega | Cat. #A6002 | - |
| 2.3 % crystal violet solution | Sigma- Aldrich | Cat. #HT901-8FOZ | 1% in 50% ethanol |
| **Service** | | | |
| DNA fingerprinting | Microsynth | - | - |
| Image Studio Lite System | Li-COR Biosciences | - | - |
