# Supplementary material for: Cisplatin-resistant A549 non-small cell lung cancer cells can be identified by increased mitochondrial mass and are sensitive to pemetrexed treatment
Source: Cancer Cell Int. 2019 Nov 29;19:317. doi: 10.1186/s12935-019-1037-1 (PMC6883680; doi:10.1186/s12935-019-1037-1)
Supplement: Supplementary file 2 — Additional file 2: Figure S1. a Working model to investigate the relationship between mitochondrial mass and chemotherapy resistance in subpopulations of the NSCLC cell line A549: We previously described three subpopulations in the parental cell line A549, e.g. holo-, mero- and paraclonal cells [16]. ChemoR (~ 100×) indicates that mesenchymal para clone cells are roughly 100 times more resistant to chemotherapy compared to holoclone cells. The question would be whether mitochondrial mass is correlated with chemoresistance (ChemoR (?x)). Within the mesenchymal paraclonal subpopulation, “A” and “B” indicate further subpopulations featuring either high or low mitochondrial mass, respectively. TICs: Tumor Initiating Cells. b Respiration measurement in A549 subpopulations and A549 Rho 0 cells: Duplex PCR products of A549 and A549 Rho 0 cells, mitochondrial DNA gene HVR (901 bp) nuclear DNA gene hNuc(467 bp). c Complex activity measurement of A549 and A549 Rho 0 cells by high-resolution respirometry OROBOROS dig, digitonin (for cell permeabilization), gm, glutamate and malate (providing nicotinamide adenine dinucleotide (NADH) to the respiratory chain complex I activation); adp, ADP (adenosine diphosphate); rot, rotenone (complex I inhibitor); succ, succinate (substrate of complex II); aa, antimycin A (Inhibitor of complex III); at, ascorbate and TMPD (N,N,N′,N′-tetramethyl-p-phenylendiamine) (substrate of Complex IV); az, sodium azide (Complex IV inhibitor). d Phase contrast images of A549 and A549 Rho 0 cells. [file 12935_2019_1037_MOESM2_ESM.pptx]

## Slide 1
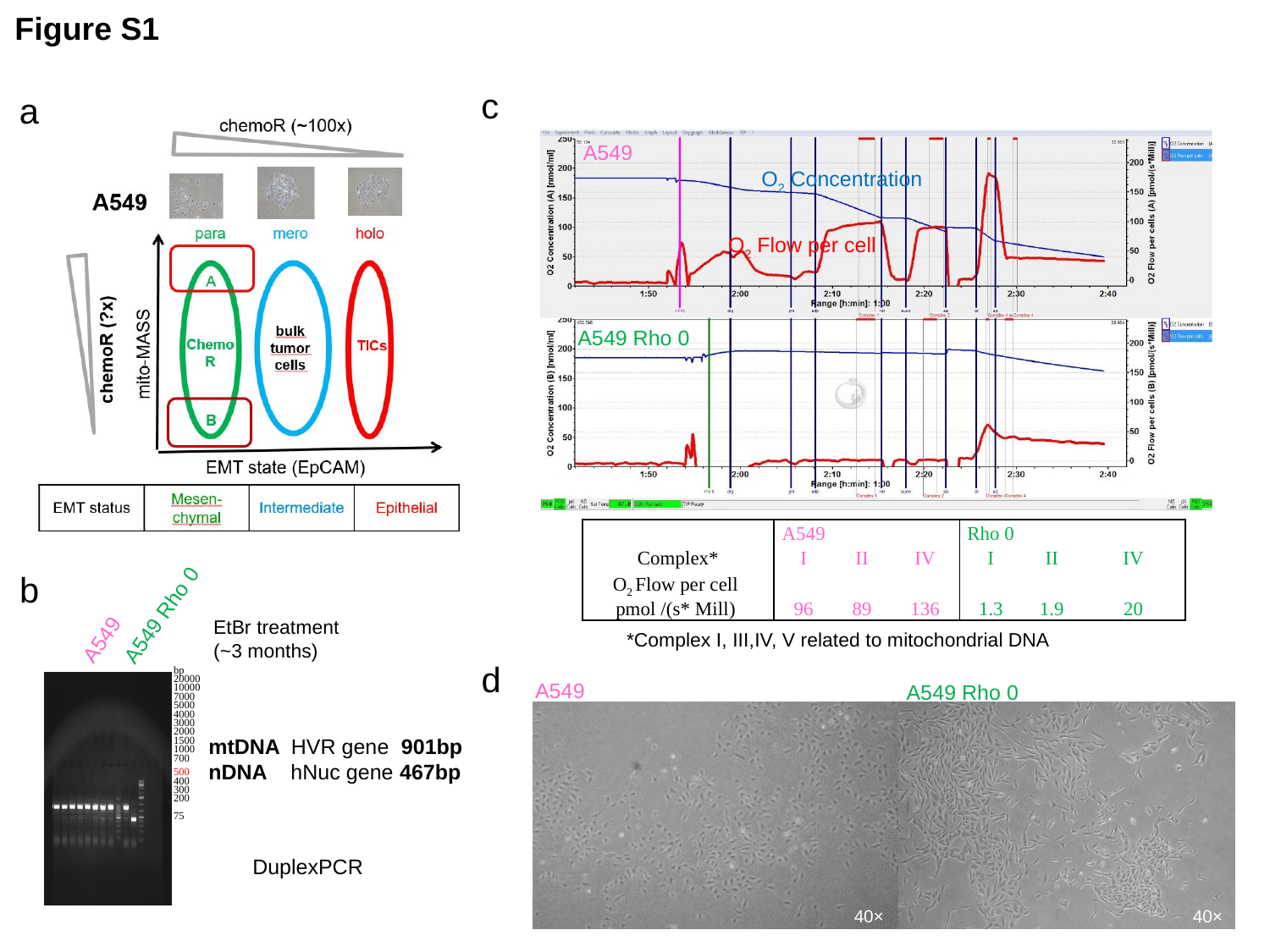

Figure S1
c
a
A549
O2 Concentration
O2 Flow per cell
A549 Rho 0
A549 Rho 0
EtBr treatment
(~3 months)
bp
20000
10000
7000
5000
4000
3000
2000
1500
1000
700
500
400
300
200
75
mtDNA HVR gene 901bp
nDNA hNuc gene 467bp
DuplexPCR
A549
| | A549 | | | Rho 0 | | |
| --- | --- | --- | --- | --- | --- | --- |
| Complex\* | I | II | IV | I | II | IV |
| O2 Flow per cell pmol /(s\* Mill) | 96 | 89 | 136 | 1.3 | 1.9 | 20 |
b
*Complex I, III,IV, V related to mitochondrial DNA
d
A549
A549 Rho 0
40×
40×
