# Supplementary figures and images for: Cisplatin-resistant A549 non-small cell lung cancer cells can be identified by increased mitochondrial mass and are sensitive to pemetrexed treatment
Source: Cancer Cell Int. 2019 Nov 29;19:317. doi: 10.1186/s12935-019-1037-1 (PMC6883680; doi:10.1186/s12935-019-1037-1)

## Slide 1
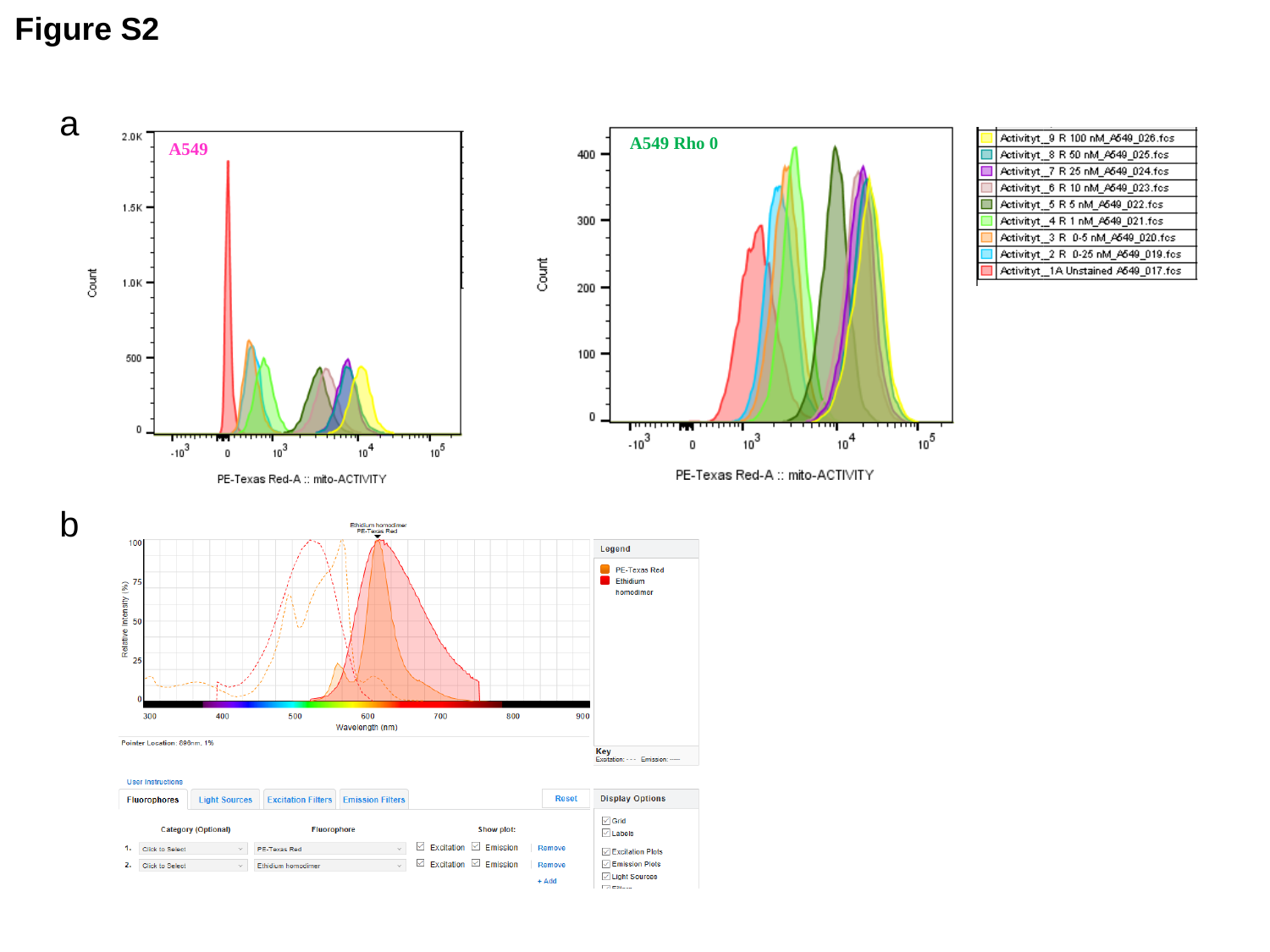

# Supplementary Figure S2
Figure S2
a
A549 Rho 0
A549
b

Supplement: Supplementary file 3 — Additional file 3: Figure S2. Unspecific MitoTracker Red CMXRos staining. a Flow cytometry analysis of A549 and A549 Rho0 cells staining with MitoTracker Red CMXRos (Mitochondrial activity dye). Different concentrations of MitoTracker Red CMXRos dye were tested, e.g. 0.25, 0.5, 1, 5, 10, 25, 50 and 100 nM. b Absorbance and emission spectra of ethidium bromide and PE-Texas Red. [file 12935_2019_1037_MOESM3_ESM.pptx]

## Slide 1
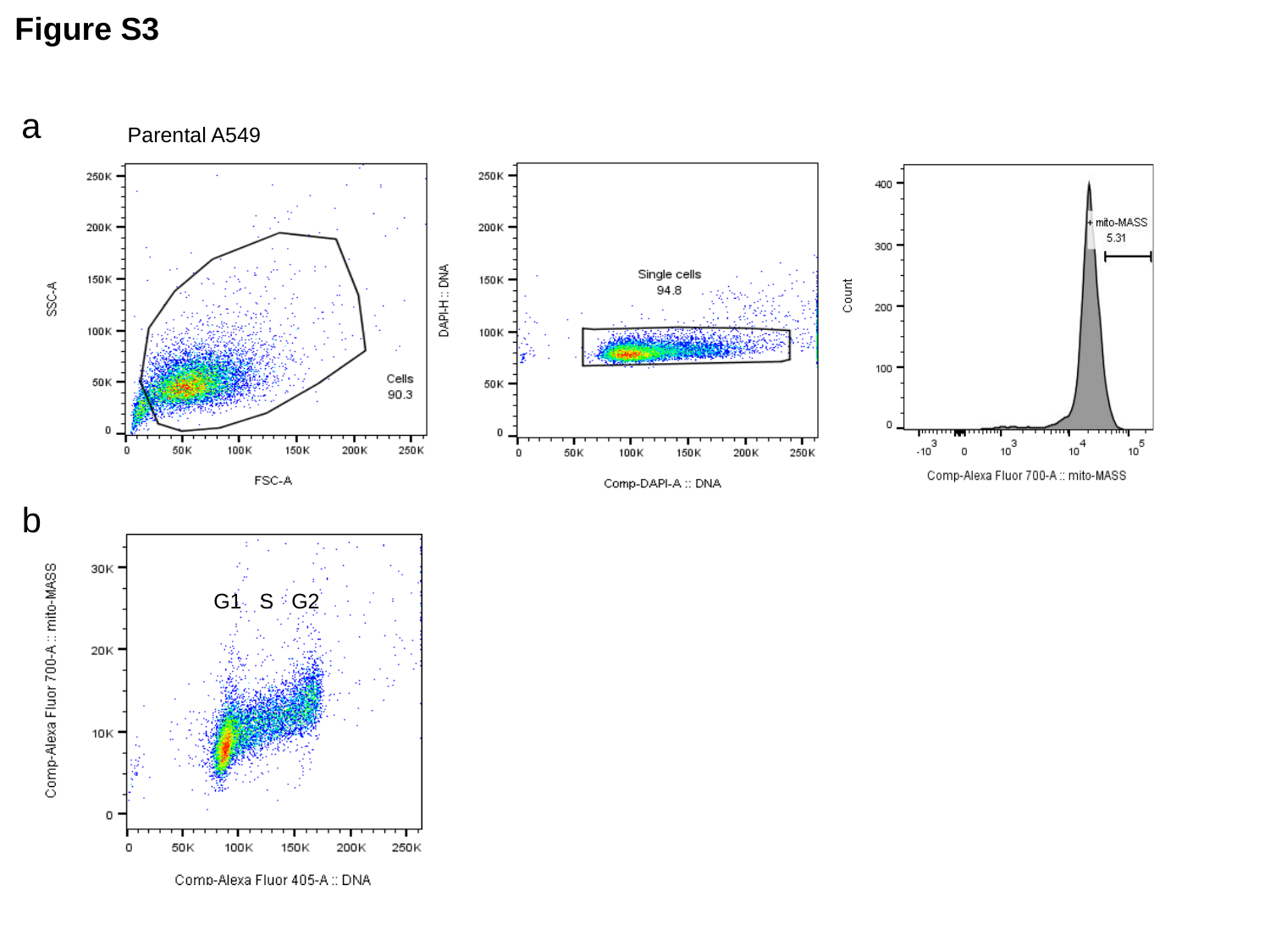

# Supplementary Figure S3
Figure S3
a
Parental A549
b
G1 S G2

Supplement: Supplementary file 4 — Additional file 4: Figure S3. Gating strategy to analyze the increase of mitochondrial mass after MTA treatment. a The gate mito-MASS+ was set as 5% in untreated A549 cells. b Mitochondrial mass distribution in different cell cycle phases of A549 cells. Mitochondrial mass of cells in G2 phase was 2 times higher comparing with G1 cells. [file 12935_2019_1037_MOESM4_ESM.pptx]
